# Plagiarism in submitted manuscripts: incidence, characteristics and optimization of screening—case study in a major specialty medical journal

**DOI:** 10.1186/s41073-016-0021-8

**Published:** 2016-10-10

**Authors:** Janet R. Higgins, Feng-Chang Lin, James P. Evans

**Affiliations:** 1 American College of Medical Genetics and Genomics, Bethesda, MD USA; 2 Department of Biostatistics, University of North Carolina at Chapel Hill, Chapel Hill, NC USA; 3 Department of Genetics, University of North Carolina at Chapel Hill, Chapel Hill, NC USA

**Keywords:** iThenticate, Plagiarism detection, Optimization

## Abstract

**Background:**

Plagiarism is common and threatens the integrity of the scientific literature. However, its detection is time consuming and difficult, presenting challenges to editors and publishers who are entrusted with ensuring the integrity of published literature.

**Methods:**

In this study, the extent of plagiarism in manuscripts submitted to a major specialty medical journal was documented. We manually curated submitted manuscripts and deemed an article contained plagiarism if one sentence had 80 % of the words copied from another published paper. Commercial plagiarism detection software was utilized and its use was optimized.

**Results:**

In 400 consecutively submitted manuscripts, 17 % of submissions contained unacceptable levels of plagiarized material with 82 % of plagiarized manuscripts submitted from countries where English was not an official language. Using the most commonly employed commercial plagiarism detection software, sensitivity and specificity were studied with regard to the generated plagiarism score. The cutoff score maximizing both sensitivity and specificity was 15 % (sensitivity 84.8 % and specificity 80.5 %).

**Conclusions:**

Plagiarism was a common occurrence among manuscripts submitted for publication to a major American specialty medical journal and most manuscripts with plagiarized material were submitted from countries in which English was not an official language. The use of commercial plagiarism detection software can be optimized by selecting a cutoff score that reflects desired sensitivity and specificity.

## Background

Plagiarism is a chronic and troublesome issue for scientific journals. It is crucial that unacceptable copying be detected to preserve the integrity of the scientific literature [1]. However, doing so places a difficult burden on editors and publishers, only partially mitigated by existing informatics approaches designed to detect plagiarism. In the past, editorial offices have essentially relied upon chance detection by reviewers or editors to discover that submitted work had been previously published. Now, due to efficient search engines, online publishing, and software algorithms, journals increasingly utilize software that can efficiently scan thousands of manuscripts in seconds, matching submitted text to already published text. Although several commercial plagiarism detection software packages exist, the majority of US publishers allow the company Turnitin to access their database of published articles. Consequently, their software, iThenticate [2], formerly called CrossCheck and also powering Similarity Check by Crossref [3], boasts that it can “prevent misconduct by comparing manuscripts against its database of over 60 billion web pages and 155 million content items, including 49 million works from more than 600 scholarly publisher participants” [2].

However, the use of iThenticate for plagiarism detection has significant limitations. iThenticate does not analyze different sections of a given manuscript (e.g., abstract and introduction), an important limitation given that some sections of manuscripts by the same group will have legitimate overlap, e.g., in the “Methods” section [4]. Indeed, both the Committee on Publication Ethics [5] and the US Office of Research Integrity [6] note that some degree of copying in this context is often legitimate. Moreover, although iThenticate can be set to ignore the bibliography and quotations, it does not always do so. Nor does iThenticate exclude title pages, affiliations, funding statements, disclosures, and acknowledgements, where original text is less important. Finally, the assessment of plagiarism is highly nuanced and reliance on a single “score” to rule unacceptable levels of copying in or out is rarely workable in practice. Though other studies have noted that journals will reject manuscripts above a certain percentage level of similarity [7], the nuances involved in differentiating actual plagiarism from legitimate overlap with previously published material mean that a single iThenticate score cannot, in practice, stand alone as a test of plagiarism. We and others [8, 9] typically use manual verification, a time-consuming and subjective approach.

The present study sought to quantify the extent of plagiarism in submitted manuscripts using iThenticate, assess whether country of origin was correlated with plagiarism, and, finally, optimize the use of iThenticate to increase efficiency of plagiarism detection in a way that balances sensitivity and specificity.

## Methods

### Manuscripts analyzed

Four hundred consecutive manuscripts were submitted to *Genetics in Medicine*, the official journal of the American College of Medical Genetics and Genomics (ACMG) from March 2013 to April 2014. We included reviews, original research, education reports, and brief reports. We excluded commentaries, documents that were generated from our society (these are usually standards and guidelines for laboratories and clinicians), and letters to the editor.

We determined the country of origin of the manuscript as inputted into our submission system by the corresponding author. From this, we checked Google as to whether English is an official language of the country.

Each manuscript, as submitted by the author, was analyzed by iThenticate with the bibliography and quotes excluded (termed full manuscript). Each manuscript was then edited to have only the abstract, introduction, results and discussion/conclusion sections. This version (termed AIRD) was then also analyzed by iThenticate with the bibliography and quotes excluded.

Each version of the manuscript that was analyzed and color coded by iThenticate was then analyzed by one author (JRH) for the following criteria:

- Only the abstract, introduction, results and discussion/conclusion sections were assessed.
- In areas highlighted by iThenticate, the sentence was deemed plagiarized if 80 % of the words in the sentence were the same as a previously published paper.
- Each sentence was scored as plagiarized or not in the section, and the number of sentences in the paragraph that were deemed plagiarized was also counted.
- Each paragraph was separately analyzed in each section; and if 80 % of the words in each paragraph were the same as a previously published paper, this was also deemed plagiarized. The number of paragraphs in each section was counted, and the number of plagiarized paragraphs was expressed as a percentage. If one sentence was deemed to be plagiarized, the manuscript was scored as plagiarized.

### Exclusion criteria

Manuscripts flagged by iThenticate were excluded from the iThenticate analysis if they were published after the manuscript was rejected or published by *Genetics in Medicine* (*GIM*).

Based on guidelines from COPE (publicationethics.org) and the US Office of Research Integrity (http://ori.hhs.gov/), we excluded the following:

- Sentences that were descriptions of standard or previously published methodologies (with appropriate referencing) or provided standard definitions of terms.
- Sentences that fell into the category of “How else would you say that?,” recognizing that in some instances almost everyone will say things virtually the same way, because the technical language involved offers little or no alternative.

As methods are the most frequently copied section (*2–4*), we did not analyze that section.

### Statistical tests

Descriptive statistics were reported by frequencies and percentages for categorical variables and by means and standard deviations for continuous variables. Two-sample comparisons were made using *t* tests for continuous variables with a normal distribution and Mann-Whitney tests for those with a non-normal distribution. Comparisons between percentages or proportions were made based on either chi-square tests or Fisher exact tests upon the number of categories. The predictability and the optimal cutoff of iThenticate score were explored using receiver operating characteristic (ROC) curve with balance between sensitivity and specificity that, respectively, calculates the successful rates of catching plagiarism if the manuscript is indeed plagiarized and claiming non-plagiarism if the manuscript is indeed a novel one. Sensitivity in terms of plagiarism detection refers, in this article, to the ability of a given iThenticate percentage score to correctly identify those manuscripts containing plagiarism when compared with the “gold standard” of manual curation (true positive rate). Specificity refers to how often an iThenticate percentage score correctly identified those manuscripts without plagiarism (reflective of the true negative rate). Area under the ROC curve (AUC) statistic was used to summarize the predictability of the iThenticate score with full texts or AIRD only. The comparison between two correlated ROC curves was made using DeLong’s test [10]. All of the statistical analyses were performed using IBM SPSS Statistics for Windows, version 23.0 (IBM Corp, Armonk, NY), and pROC package in R 3.2.3 (R Foundation for Statistical Computing, Vienna, Austria). *p* values <0.05 were considered statistically significant.

## Results

Four hundred consecutively submitted manuscripts to *GIM*, the official journal of the American College of Medical Genetics and Genomics, were manually curated for plagiarism (see the “Methods” section for description) and analyzed by iThenticate. Manuscripts were scored by country of origin and whether English was an official language of that country.

Of 400 manuscripts analyzed, 357 were original research articles; there were 43 review manuscripts. One manuscript was excluded on the basis that it was an adaptation of a report for an agency and subsequently written as a review for *GIM* (the manuscript was ultimately withdrawn by the authors). Final analysis was therefore performed on 399 manuscripts.

**Fig. 1:**
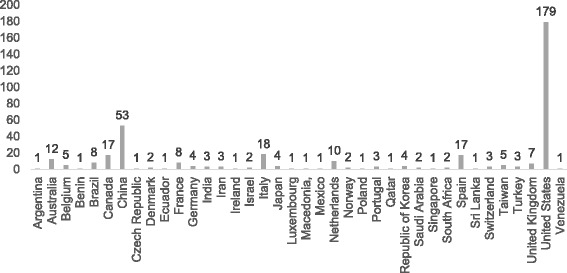
Manuscripts by country of origin

Figure 1 shows manuscripts by country of origin. English was an official language in 232 manuscripts (58 % of all manuscripts being analyzed) from 9 countries versus 30 countries where English was not an official language.

Manual curation determined that 66 manuscripts (17 %) contained plagiarized material. Of these 66, 55 (82 %) came from countries where English was not an official language (*p* < 0.001 Fisher’s exact test compared to countries where English was an official language). Table 1 shows country of origin of the manuscripts with plagiarism.

**Table 1:** Country of origin of the manuscripts with plagiarism

Country	Manuscripts with plagiarism (%)	iThenticate score median (range)^a^	Self-plagiarism (%)
Brazil	3 (38)	25 (17–38)	2 (67)
China	23 (43)	29 (10–53)	10 (43)
France	1 (12)		1 (100)
India	1 (33)		0
Islamic republic of Iran	2 (67)		1 (50)
Italy	6 (33)	26 (20–37)	4 (67)
Japan	2 (50)		0
Macedonia	1 (100)		0
Netherlands	1 (10)		1 (100)
Norway	1 (50)		1 (100)
Portugal	1 (33)		1 (100)
Republic of Korea	1 (25)		1 (100)
Spain	6 (35)	24.5 (18–33)	5 (83)
Sri Lanka	1 (100)		0
Taiwan	3 (60)	26 (19–43)	1 (33)
Turkey	3 (100)	32 (10–44)	1 (33)
United States	10 (6)	17 (9–28)	6 (60)
Grand total	66		35

^a^Given for *n* > 3 only

Among submissions from countries in which English is not an official language, China had the highest levels of plagiarism: 23/67 (34 %). Where English is an official language, manuscripts most often came from the USA (10/11; 91 %) (Table 1).

### Time spent

For manuscripts that were deemed to have plagiarized material, it took on average 5.9 min (median = 5 min) to manually assess the manuscript (range 2–20 min). For manuscripts deemed not to have plagiarism, it took on average 1 min to manually assess the manuscript (range 1–5 min), a statistically significant difference (*p* < 0.001, Mann-Whitney test).

### iThenticate

Among the 66 manuscripts deemed to have plagiarism by manual curation of the full manuscript, the average iThenticate score was 25.8 (range 9–53, SD = 9.9), compared with an average score of 11.5 (range, 0–39, SD = 6.2) from 333 manuscripts deemed not to have plagiarism by manual curation (*t* test *p* value <0.001).

When manuscripts were edited to only include abstract, introduction, results, and discussion (AIRD), those deemed to have plagiarism by manual curation still had a similar iThenticate score (mean = 25.7, range 1–59, sd = 14.9), compared with a much lower iThenticate score in manuscripts deemed not to have plagiarism by manual curation (mean = 5.6, range 0–25, sd = 5.0). The difference again reached statistical significance (*t* test *p* value <0.001).

### Optimizing the use of iThenticate to efficiently predict plagiarism

**Fig. 2:**
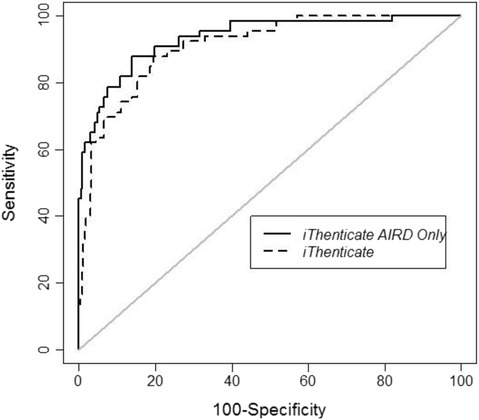
Receiver operating characteristics (ROC) of iThenticate scores

Using manual curation as the gold standard for plagiarism detection, we sought to determine the performance of iThenticate to detect it. We performed an ROC analysis to maximize the sensitivity and specificity of iThenticate scores (Fig. 2). For both AIRD and the full manuscripts, iThenticate scores had a good classification rate, with the AIRD area under the curve (AUC) being 0.928 (95 % CI, 0.891–0.9644), and the full manuscript iThenticate score AUC being 0.902 (95 % CI, 0.863–0.940), a non-significant difference (*p* = 0.122). Thus, since analysis of either AIRD or full manuscripts yielded equivalent scores, we focused on iThenticate scoring of full manuscripts to minimize the amount of time necessary to edit a manuscript to AIRD. The cutoff score maximizing both sensitivity and specificity was 15 % (Table 2). In other words, using an iThenticate score of 15 % as a cutoff would have successfully “caught” 84.8 % of plagiarized manuscripts (sensitivity) with a specificity of 80.5 % (Table 3). Since in some contexts, users might desire a higher sensitivity (and accept lower specificity), we generated a table reflecting a range of sensitivity values with corresponding specificities to allow tailoring of cutoff scores (Table 2).

**Table 2:** Sensitivity and specificity as a function of iThenticate scores

iThenticate—overall similarity %	Sensitivity	Specificity	Sensitivity + specificity
0	1.000	0.003	1.003
2	1.000	0.018	1.018
3	1.000	0.045	1.045
4	1.000	0.096	1.096
5	1.000	0.156	1.156
6	1.000	0.201	1.201
7	1.000	0.276	1.276
8	1.000	0.333	1.333
9	0.985	0.429	1.414
10	0.955	0.483	1.438
11	0.939	0.559	1.498
12	0.924	0.670	1.594
13	0.894	0.727	1.621
14	0.879	0.769	1.648
*15*	*0.848*	*0.805*	*1.653*
16	0.818	0.814	1.632
17	0.758	0.847	1.604
18	0.742	0.862	1.604
19	0.712	0.889	1.601
20	0.697	0.907	1.604
21	0.697	0.919	1.616
22	0.636	0.934	1.570
23	0.621	0.955	1.576
24	0.561	0.967	1.528
25	0.470	0.967	1.437
26	0.394	0.970	1.364
27	0.364	0.982	1.346
28	0.318	0.985	1.303
29	0.303	0.988	1.291
30	0.258	0.988	1.246
31	0.242	0.988	1.230
32	0.197	0.991	1.188
33	0.167	0.991	1.158
34	0.167	0.994	1.161
35	0.167	0.997	1.164
37	0.152	0.997	1.149
38	0.136	0.997	1.133
39	0.121	1.000	1.121
40	0.106	1.000	1.106
41	0.091	1.000	1.091
42	0.076	1.000	1.076
43	0.061	1.000	1.061
44	0.045	1.000	1.045
45	0.030	1.000	1.030
48	0.015	1.000	1.015
53	0.000	1.000	1.000

Italic values indicate the score that maximized both sensitivity (at 85 %) and specificity (at 80 %)

**Table 3:** Sensitivity and specificity calculations using an iThenticate score of 15 % compared to manually detected plagiarism

		Manual curation (gold standard)	
		No plagiarism	Plagiarism
iThenticate overall similarity score 15 %	No plagiarism	268 (80.5 %)	10 (15.2 %)
	Plagiarism	65 (19.5 %)	56 (84.8 %)
	Total	333	66

The optimal iThenticate score is 15 % where the sensitivity is 84.8 % (66 manuscripts had plagiarism by manual curation and iThenticate correctly identified 56 of those manuscripts) and the specificity is 80.5 % (333 manuscripts had no plagiarism by manual curation and iThenticate correctly identified no plagiarism in 268 of these manuscripts)

### Final disposition of manuscripts

Since integration of iThenticate into our system in 2012, the policy at *GIM* has been to screen all manuscripts with iThenticate just prior to acceptance. As this study was retrospective, many of the manuscripts had previously been screened for plagiarism but using a much less systematic approach to detecting plagiarism than applied during the study. Of the 66 manuscripts deemed in this study to have plagiarism, we retrospectively looked to determine those manuscripts’ final disposition: we rejected 54 without review and rejected 3 after review so the plagiarism was not an issue. To determine the ultimate fate of those 57 manuscripts, we searched Google and PubMed and found 37/57 (65 %) had indeed been published elsewhere, 18 of the 37 in open access journals. In 34/37 of the plagiarized manuscripts rejected by *Genetics in Medicine* and published elsewhere, almost exactly the same text was found, suggesting that the authors simply submitted their article to another journal and that journal either did not check for plagiarism or did not deem the plagiarism to rise to a level of concern.

Nine subsequently found to have unacceptable levels of plagiarism were accepted and published by *GIM*. Examining the history of each of the nine accepted manuscripts; four were deemed at the time of acceptance to have some overlap with previously published text from the same authors and the authors were asked to rewrite portions of the text before publication; two were deemed to be in the grey area of “how else could you say that?”; three were published with one paragraph similar to previously published papers.

In no manuscripts was there deemed to be any data copying or reuse.

### Self-plagiarism

We analyzed the iThenticate report for each manuscript deemed to have plagiarism by manual curation, to determine if the text copying was reuse of authors’ own previously published articles (i.e., self-plagiarism). The iThenticate report lists all articles and authors that match the copied text in the submitted manuscript. From this list we searched whether the authors had used their own previously published articles to copy text, and if any article matched the co-author list we deem this self-plagiarism. 35/66 (53 %) were considered self-plagiarism (Table 1 for a breakdown by country).

### Implementation of Chinese language instructions to authors

This study revealed that approximately half of copying was text reuse from others and about half was copied from authors’ own published work. It also revealed that authors from China were most likely to copy text. In November 2014 we introduced Chinese language instructions for authors (IFA) that specifically addressed ethics and plagiarism, to determine if such education of authors could reduce text copying in submitted manuscripts. We then analyzed consecutive manuscripts from the USA, Spain and China submitted between November 2014 and June 2015. Manuscripts from USA and Spain were chosen as control countries, as they had both had relatively high levels of text copying in our initial study (see Table 1) but should be unaffected by the Chinese language IFA. Manuscripts were manually curated as well as analyzed by iThenticate. There was no change in the levels of detectable text plagiarism in manuscripts submitted from Spain, USA or China (Table 4).

**Table 4:** Plagiarism in USA, Spain, and China manuscripts before and after implementation of Chinese language instructions for authors

		Before implementation of Chinese language IFA		After implementation of Chinese language IFA	
		No plagiarism	Plagiarism	No plagiarism	Plagiarism
Manuscripts country of origin	China	30 (14.3 %)	23 (59.0 %)	18 (11.1 %)	13 (52.0 %)
	Spain	11 (5.2 %)	6 (15.4 %)	12 (7.4 %)	2 (8.0 %)
	United States	169 (80.5 %)	10 (25.6 %)	132 (81.5 %)	10 (40.0 %)
	Total	210	39	162	25

This table shows the column percentages of plagiarism in three countries before and after the implementation of Chinese language instructions to authors (IFA). There was no significant reduction in plagiarism, detected by manual curation, in any of the three countries analyzed (chi-square test, *p* = 0.821)

Among manuscripts from China, 43.4 % (23/53) had plagiarism before the implementation of Chinese language IFA versus 41.9 % (13/31) after implementation of IFA (Fisher exact test p = 1.000). Neither Spain nor the USA showed significant changes in plagiarism with the introduction of Chinese language IFA. Thus, we found no evidence that Chinese language IFA had a significant impact on reducing the likelihood of plagiarism (chi-square test *p* value = 0.821).

## Discussion

Plagiarism is common and threatens the integrity of the scientific literature. However, detection of plagiarism is time consuming and difficult. In this study we document the extent of plagiarism in submissions to a major medical journal, derive flexible criteria that can be used to optimize the most common plagiarism detection software and demonstrate that a disproportionate amount of plagiarized manuscripts come from specific countries in which English is not a native language, most notably, China.

As have others before us [11, 12], we find iThenticate to be a useful tool for alerting editors to possible plagiarism. However without manual curation, it is impossible to determine if text copying rises to the level of plagiarism. Studies we found using iThenticate were vague in how plagiarism was determined, e.g., “…2 individuals (Editor-in-Chief, Managing Editor) separately assessed the duplications and rated them as being significant or insignificant” [12] and there are few quantitative studies of plagiarism in scientific manuscripts. Bazdaric [9] et al. used a criteria of 10 % or more similar text with one source in CrossCheck (iThenticate) to identify plagiarism and in the abstract, those authors used a threshold of 6 consecutive words to define plagiarism. However, in an effort to keep the definition simple, we chose a relatively strict criteria for assessment of plagiarism, namely 80 % of a sentence being copied. We also hoped this would avoid non-specificity as 6 consecutive words would regularly pick up simple disease descriptions (for example “Cystic fibrosis is an inherited disease”), thus being highly sensitive but insufficiently specific.

This study found an unacceptable level of text plagiarism in 17 % of articles submitted to *GIM*, which was unexpectedly high. As expected, analysis of these manuscripts by iThenticate resulted in a score that was significantly higher than in manuscripts deemed not to have plagiarism. Importantly, this was true whether the manuscript was edited only to contain the abstract, introduction, results and discussion/conclusions (AIRD), removing extraneous text that may confound iThenticate. Reassuringly, there were no instances of data reuse/copying in this subset. Just over half of the manuscripts with plagiarism were “self-plagiarized”. This is an area of current ethical debate on websites such as COPE who have renamed this type of copying “text recycling.” Regardless, there is general consensus that self-plagiarism is should be avoided [13–15].

We wished to optimize the use of iThenticate, performing ROC analysis to generate a Table of iThenticate scores with specificity and sensitivity (Table 2). For example, an iThenticate score of 15 % results in a sensitivity of 85 % and specificity of 80 % for plagiarism detection. This threshold is in line with users of Turnitin software [16, 17], which is also produced by Turnitin and compares papers of K-12 and college students to papers/text already published. Importantly, this table allows users to adjust sensitivity and specificity to optimize use of iThenticate for a particular context. For example, if one is willing to tolerate lower specificity, a cutoff of eight would detect all plagiarized manuscripts submitted, but one in three manuscripts would a false positive.

Manuscripts from countries where English is not an official language had a significantly higher rate of plagiarism; a finding determined by other studies [9, 18]. Authors from China submitted the highest number of plagiarized manuscripts, with Spain (6/17) and Italy (6/18) the next most frequent. For countries where English is an official language, the United States was the highest with 10 manuscripts, but this only accounted for 2 % of US submissions.

One limitation of this study was reliance on author’s designation of country of origin. This may be more truly an indication of where the work was performed rather than the author being a native English speaker. However, we felt this assumption justified since it is likely that for manuscripts from a country where English was an official language at least one co-author would be a native English speaker. Moreover, assignment of plagiarism is, ultimately somewhat subjective and there exists no consensus in the editorial community. Thus, we chose one reasonable set of criteria but, of course, specific definitions might vary. Therefore, it is critical that we provide here a set of cutoff scores that users can adjust in different contexts.

**Fig. 3:**
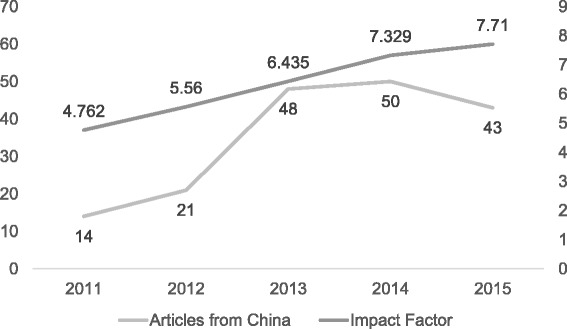
GIM’s impact factor and number of manuscripts submitted from China

Publications from China have risen over the last several years [19], likely in part because Chinese scholars are required to publish in English language journals as part of their degree and/or promotion requirements [20]. There are often financial rewards for doing so [20] and the higher the impact factor of the journal the higher the cash incentive [21]. We noted an increase in submissions from China when *GIM’s* impact factor rose above 5 (Fig. 3). It has also been shown that learning by copying verbatim is more common in China [22] and it has been noted that for Chinese students using another author’s words is a sign of respect [23]. However, the issue of culture and plagiarism is nuanced, and as Pecorari and Petric state “a more fruitful enterprise may be studying [the students] plagiarism for what it can teach us about their experiences as writers.” [24].

We attempted to reduce plagiarism in manuscripts submitted from China by implementing a set of instructions to authors in Chinese that contained a section on ethics (http://www.nature.com/gim/gim_gta_chinese.pdf). But this educational tool was ineffective. Previous studies disagree on whether merely warning authors that plagiarism is actively looked for is an effective deterrent [25, 26] and it may require more active educational efforts on these topics [24, 25]. Both increased education as well as facile mechanisms for finding plagiarism are clearly needed to reduce this threat to the academic literature.

## Conclusions

Plagiarism was a common occurrence among manuscripts submitted for publication to *Genetics in Medicine*, a major American specialty medical journal. Most manuscripts with plagiarized material were submitted from countries in which English was not an official language, most notably China. Using the sensitivity and specificity table generated in this work, the use of iThenticate commercial plagiarism detection software can be tailored to specific uses with selection of an appropriate cutoff score that maximizes either sensitivity or specificity.
